# Biogeography and Potential Exchanges Among the Atlantic Equatorial Belt Cold-Seep Faunas

**DOI:** 10.1371/journal.pone.0011967

**Published:** 2010-08-05

**Authors:** Karine Olu, Erik E. Cordes, Charles R. Fisher, James M. Brooks, Myriam Sibuet, Daniel Desbruyères

**Affiliations:** 1 Département Etude des Ecosystèmes Profonds (DEEP), IFREMER, BP70, 29280 Plouzané, France; 2 Biology Department, Temple University, Philadelphia, Pennsylvania, United States of America; 3 Biology Department, Pennsylvania State University, University Park, Pennsylvania, United States of America; 4 TDI-Brooks International, College Station, Texas, United States of America; 5 Institut Océanographique, Paris, France; Paleontological Institute, Russian Federation

## Abstract

Like hydrothermal vents along oceanic ridges, cold seeps are patchy and isolated ecosystems along continental margins, extending from bathyal to abyssal depths. The Atlantic Equatorial Belt (AEB), from the Gulf of Mexico to the Gulf of Guinea, was one focus of the Census of Marine Life ChEss (Chemosynthetic Ecosystems) program to study biogeography of seep and vent fauna. We present a review and analysis of collections from five seep regions along the AEB: the Gulf of Mexico where extensive faunal sampling has been conducted from 400 to 3300m, the Barbados accretionary prism, the Blake ridge diapir, and in the Eastern Atlantic from the Congo and Gabon margins and the recently explored Nigeria margin. Of the 72 taxa identified at the species level, a total of 9 species or species complexes are identified as amphi-Atlantic. Similarity analyses based on both Bray Curtis and Hellinger distances among 9 faunal collections, and principal component analysis based on presence/absence of megafauna species at these sites, suggest that within the AEB seep megafauna community structure is influenced primarily by depth rather than by geographic distance. Depth segregation is observed between 1000 and 2000m, with the middle slope sites either grouped with those deeper than 2000m or with the shallower sites. The highest level of community similarity was found between the seeps of the Florida escarpment and Congo margin. In the western Atlantic, the highest degree of similarity is observed between the shallowest sites of the Barbados prism and of the Louisiana slope. The high number of amphi-atlantic cold-seep species that do not cluster according to biogeographic regions, and the importance of depth in structuring AEB cold-seep communities are the major conclusions of this study. The hydrothermal vent sites along the Mid Atlantic Ridge (MAR) did not appear as “stepping stones” for dispersal of the AEB seep fauna, however, the south MAR and off axis regions should be further explored to more fully test this hypothesis.

## Introduction

Since the discovery of lush communities of specialized animals associated with deep-sea vents [1] and cold-seeps [2] the question of biogeography of the inhabitants of these isolated chemosynthesis-based ecosystems has been one of the major persistent questions [3], [4], [5], [6], [7], [8], [9], [10], [11], [12]. Studies of the hydrothermal vent fauna have defined several biogeographic provinces (EPR, Northern Pacific, MAR…) based on faunal composition and patterns of endemicity which are consistent with historical geological events. The latest analysis, which included hydrothermal vent fauna from 63 vent fields, supported the presence of 6 major biogeographic provinces [13]. Meanwhile cold seeps have been discovered worldwide along continental margins and have also been grouped into several biogeographic provinces (Gulf of Mexico, Atlantic, Mediterranean, East Pacific and West Pacific) [9]. However, high estimated rates of gene flow among disjunct populations of various species (reviewed by [14], [15], [16] and the genetic similarity of several groups of widely distributed vent and seep endemic taxa [10], [17] suggest high capacities of dispersal within and potentially among these seep and vent biogeographic provinces. One striking example is the siboglinid tubeworm *Escarpia spicata* Jones, 1985, which inhabits cold seeps and whale falls off southern California, seeps on the Pacific margin of Costa Rica and sedimented hydrothermal vents in the Gulf of California, which is thus-far genetically indistinguishable from *Escarpia laminata* Jones, 1985 living at cold seeps in the Gulf of Mexico [18], [19] and *Escarpia southwardae* Andersen et al. 2004, from the Gulf of Guinea [20]. Reproductive strategies may explain high dispersal capacities for some species, as for the seep mytilid *Bathymodilus childressi* Gustafson et al., 1998, which have broadcast spawning and produce numerous long-lived planktotrophic larvae [21], [22]. The high number of seep sites may also favour dispersal along continental margins of non planktotrophic taxa such as vesicomyid bivalves [23].

Depth has also been shown to influence the distribution of vent and seep organisms, as it has for the general deep-sea fauna in all ocean basins along margins [24]. Difference in depth has been hypothesized as a barrier to successful colonization of organisms along the Mid-Atlantic Ridge [3], [25] and in seeps on continental margins [5], [12], [26], [27], [28].

Luxurious cold-seep communities are known from both sides of the equatorial Atlantic ocean between 32°29′N (Blake Ridge) and 05°47′S latitude (Regab pockmark) and between 350m and 3300m depth. They have been extensively studied in the northern Gulf of Mexico, a broad area of hydrocarbon seepage resulting from salt tectonics. The first seep communities were described from the Florida Escarpment at 3300 m depth [2], [29], and others were soon discovered at shallower depths [30], [31], [32]. Numerous additional seep sites were discovered and more detailed community-level characterizations followed, both on the upper slope above 800 m [2], [33], [34], [35], [36], [37], [38], [39] and the lower slope at greater depths [12], [40], [41], [42]. Cold-seep communities in the western Atlantic have also been described from a few dives on mud volcanoes and diapirs between 1000 and 5000m depth in the Barbados accretionary prism area [43], [44], [45] and from the Blake ridge diapir off North Carolina [46]. More recently seep communities have been discovered in the eastern Atlantic, on a giant pockmark cluster in the Gulf of Guinea near the Congo deep channel [47], [48], also on other pockmarks of the Congo margin [49], Gabon margin [50] and Nigeria margin [12] and in the Gulf of Cadiz [51]. All revealed dense invertebrate communities associated with mytilid mussels, vesicomyid clams, and/or siboglinid tubeworm aggregations. The similarities in landscape, habitat and dominant taxa of many of these cold-seep sites located on both sides of the Atlantic ocean led to the selection of the Atlantic equatorial belt (AEB) as an area to more closely examine the biogeography of deep chemosynthetic ecosystems in the Census of Marine Life ChEss project [9].

The first taxonomic investigations provided evidence for strong affinities at least at the genus level between the cold-seep fauna of the Barbados seeps in the Caribbean region and Gulf of Mexico [45], the Blake ridge and Florida escarpment [46], and more recently between Congo and west Atlantic equatorial cold seeps [47]. A comparison of tubeworms, mussels, and associated megafauna communities among sites of the different West Atlantic seep sites, suggested a broadly distributed community structured primarily by depth rather than by distance [12]. Hypotheses of recent genetic connections among seep taxa on the two sides of the Atlantic ocean were confirmed by genetic studies of Bathymodiolinae mussels, which revealed a high degree of genetic similarity between two species from Nigerian margin cold seeps and *B. childressi* and *Bathymodiolus heckerae* Gustafson et al., 1998, from the Gulf of Mexico ([12]). A study combining morphology and genetics supported two species complexes of amphi-Atlantic cold-seep mussels [52]: a first including *Bathymodiolus boomerang* Cosel & Olu, 1998 from Barbados, *B. heckerae* from the Gulf of Mexico, and *Bathymodiolus* aff. *boomerang* from the Gulf of Guinea, and a second one, the *B. childressi* complex, including species from the Louisiana slope, Barbados, Congo and Nigerian margin seeps. Another species from the Gulf of Cadiz attributed to *Bathymodiolus mauritanicus* Cosel, 2002 may belong to the *B. childressi* complex [51]. There also appears to be segregation by depth of these complexes, with species of the *B. boomerang* complex living deeper than those of the *B. childressi* complex [52].

In this paper we review the recent studies of fauna inhabiting West and East Atlantic cold seeps and present a new analysis of similarity among faunal collections, which includes a large number of additional Gulf of Mexico seep sites and sites in the Gulf of Guinea. In this intra- and inter-regional comparison, depth and geographic distance are compared as factors influencing large-scale distribution of seep species. Other abiotic and biotic factors likely to influence the structure of seep communities and the dispersal capacity of the deep chemosynthetic fauna are discussed, and further exploration of potential dispersal “stepping stones” are proposed.

## Methods

**Figure 1:**
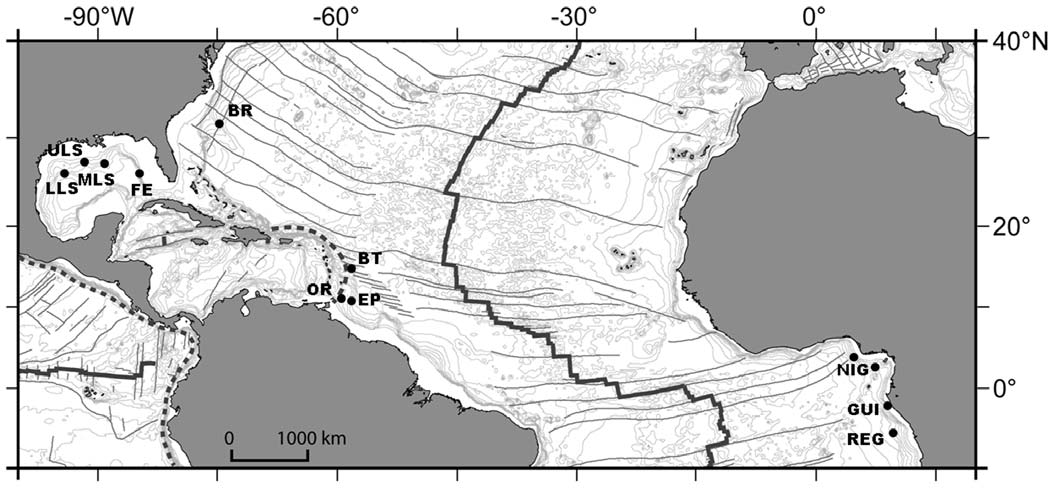
Locations of known cold-seep sites along the Atlantic Equatorial Belt. Gulf of Mexico: ULS, MLS, LLS: upper, middle and lower Louisiana slope, FE: Florida Escarpment; BR: Blake Ridge diapir; Barbados prism: OR: Orenoque A & B sectors, EP: El Pilar sector, BT: Barbados trench; Gulf of Guinea: REG: Regab pockmark, GUI: Guiness area, NIG: Nigerian slope. For depths and references refer to Table 1. The Nigerian slope sites have been only partially sampled.

A list of taxa was established from a literature review of Equatorial Atlantic cold-seep community composition [6], [12], [34], [36], [37], [40], [42], [44], [45], [46], [47], [49], [53] and taxonomic papers [52], [54], [55], [56], [57], [58], [59], [60]. The AEB cold-seep sites explored up to now form 4 regions: the Gulf of Mexico, the Barbados prism, the North Carolina margin (Blake Ridge) and the Gulf of Guinea (Fig. 1; Table 1). Because of many cold-seep sites sampled along the Louisiana Slope, it was divided into upper slope (<1000 m), mid-slope (1000–2000 m), and lower slope (2000–3000 m), while the Florida Escarpment sites are at 3200m depth. Records from the Nigerian margin where cold seeps were sampled by box cores by both American [12] and French teams (unpubl.) were included in the list of taxa but not in the statistical analyses due to limited sampling effort and absence of submersible dives. We also omitted in some later analyses the Guiness site off Gabon (600m depth), and the Barbados trench mud volcanoes (4900m depth), although explored and sampled by ROV, because of the absence at both site of any known shared species with any of other Atlantic sites.

**Table 1:** Cold-seep sites included in this study.

Region	Site	Abbreviation	Latitude (mean)	Longitude (mean)	Depth (mean)	References
Gulf of Mexico	Louisiana Upper Continental Slope	ULS	27°44′N	91°16′W	550m	Bergquist et al. 2003, Cordes et al. 2005
Gulf of Mexico	Louisiana Middle Continental Slope	MLS	27°06′N	91°10W	1500m	Cordes et al. in press
Gulf of Mexico	Louisiana Lower Continental Slope	LLS	26°21′N	94°30′W	2300m	Brooks et al. 1990, Cordes et al. 2007, Cordes et al. in press
Gulf of Mexico	Florida Escarpment	FE	26°01′N	84°55′W	3300m	Paull et al. 1984, Turnipseed et al. 2003, Cordes et al. 2007
NW Atlantic	Carolina margin Blake Ridge	BR	32°30′N	76°11′W	2155m	Van Dover et al. 2003
Barbados Prism	North Barbados Trench	BT	13.8333	−57.6666	4850m	Olu et al. 1997
Barbados Prism	South Barbados Orenoque A	OA	10°21′N	58°51′W	1700m	Jollivet et al. 1990, Olu et al. 1996
Barbados Prism	South Barbados Orenoque B	OB	10°19°	58°37	2000m	Olu et al. 1996
Barbados Prism	South Barbados El Pilar	EP	11°13N	59°21W	1200m	Olu et al. 1996
Gulf of Guinea	Congo basin Regab	Regab	05°46.89′S	09°44.66′E	3170m	Olu-le Roy et al.2007, Olu et al. 2009
Gulf of Guinea	Congo basin Astrid	Ast	4°57′S	10°09.5′E	2830m	Olu et al. unpubl.
Gulf of Guinea	Congo basin,	HH, BH, WH	04°49′S-04°46′S	9°54′E–9°56′E	3100m	Sahling et al. 2008
Gulf of Guinea	Gabon margin, Guiness	Gui	1°34.64′S	8°32.90′E	680m	Olu et al. unpubl.
Gulf of Guinea	Nigeria margin	NIG	4°59′N	4°08′E	1350m	Cordes et al. 2007
Gulf of Guinea	Nigeria margin	NIG	2°58′N	6°38′E	1600m	Galéron, Olu, et al. unpubl.

The underlined sites are included in the similarity and principal component analyses.

Because of large differences in sampling efforts among sites, in particular between Barbados and Gulf of Mexico sites, only sampled megafaunal taxa, and bivalve commensals were included in statistical analyses (comparable to [12]). Only taxa identified to the species level (named or known as new species) were included; e.g. we omitted records as *Lamellibrachia* sp. or *Escarpia* sp. that were reported from 3 different sites of the GoM or Barbados, but used *Calyptogena* cf. *kaikoi* from Barbados trench based on molecular comparisons to specimens from the Florida escarpment and Mid-Atlantic Ridge [17]. The species complexes of Bathymodiolinae were considered as a single record (as a species) in the similarity analyses. Finally, the taxa morphologically indistinguishable from described species, but lacking molecular confirmation of their identity, were reported as *affinis* (e.g. *Phymorhynchus cingulatus*, *Chiridota heveva*) and considered to be the same species for these analyses. A total of 72 species (19 symbiont-bearing and 53 associated species) were associated in the multivariate analyses. Similarity between sites based on their faunal composition was analysed using clustering analysis and ordination (PCA). Nine sites were included in this analysis (Table 1). In the Gulf of Guinea, only the REGAB pockmark and those from the GUINESS area were sufficiently surveyed and sampled to be compared with the other sites. The Barbados trench mud volcanoes (BT) and the Guiness pockmarks (Gui) from the Gabonese margin were excluded as sharing only one (Gui) or no (BT) species with the other sites. Their inclusion in the dataset do not change the relationships among the other sites in both Similarity and PCA analyses. Both Hellinger distance and Bray-Curtis similarity, based on species presence-absence data, were used for the cluster analysis based on “Ward” linkage. Ward's method, like the Group-Average method, is an agglomerative technique that uses the squared Euclidean Distance (or here the Hellinger distance) measured between the cluster centroids [61], [62]. The PCA ordination represents Hellinger distance based on species presence-absence data.
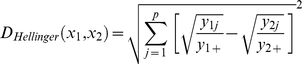
The Hellinger distance is another technique for clustering or ordination of species abundance data that allows representation in an ordination space that conserves metric distances and does not consider double absence as an indicator of similarity between samples [63]. All analyses were performed using the Vegan package in R [64].

## Results

### Variability among cold-seep communities in the Gulf of Guinea

Preliminary investigations along the African Margin from 600 to 3300m depth and along Congo-Angola, Gabon and Nigeria margins, have revealed a relatively high degree of dissimilarity among the different areas investigated, which further demonstrates the variability of seep communities at this spatial scale (100–1000 km). The Astrid pockmark, and other pockmarks described from the Congo basin around 3000m depth (Hydrate Hole, Black Hole and Worm Hole) were explored by only one ROV dive or TV-guided grab each but revealed similarities in the largest megafauna, symbiont-bearing (vesicomyids and mytilid bivalves, escarpid tubeworms) or associated taxa (alvinocarid shrimps, galatheids and synaptid holothurids) within this small area (Table S1). The Guiness pockmarks located at 650 m depth along the Gabon margin are characterised by low seep emissions and are colonised only by patchy beds of small vesicomyids. The associated megafauna consist of a small number of species compared to the fauna associated with the different habitats of the other pockmark sites. The Nigerian margin cold seeps have not been yet comprehensively sampled, but seem to have higher similarity to the Regab site off Congo than to the Guiness pockmarks. One striking peculiarity of the Nigeria slope compared to other known African sites is the presence of large Cladorhizidae sponges, resembling to those of the Barbados trench seeps.

### Comparison among seep communities in the Atlantic equatorial belt

The highest similarities among the regions encompassed by the AEB, in terms number of shared megafauna species, were found in the west Atlantic between the Gulf of Mexico and the Barbados prism with 10 species found so far, followed by the Gulf of Mexico and Gulf of Guinea with 8 or 9 species (Tables 2, 3). The Blake Ridge diapir showed a lower similarity with other regions but affinities with all three other regions. The highest number of shared taxa between two sites of different regions was found between east and west Atlantic cold seep sites of the Florida escarpment and Regab pockmark, with 7 shared species identified so far (Table 2), and a high level of similarity in the families of polychaetes at the two sites (Table S1). Nigerian margin seeps also may have a high degree of similarity with West Atlantic seeps (Table S1). In the Gulf of Guinea, the shallow and less active Guiness pockmark communities appeared very different from those of the deep Regab site and did not share any species with other AEB cold-seep sites.

**Table 2:** Numbers of shared species (in bold) and Bray-Curtis distances.

	ULS	MLS	LLS	FE	EP	OA	OB	BT	BR	Reg	Gui
ULS	**26**	**9**	**4**	**1**	**3**	**4**	**2**	**0**	**0**	**1**	**0**
MLS	0.59	**18**	**11**	**7**	**2**	**3**	**3**	**0**	**3**	**2**	**0**
LLS	0.83	0.44	**21**	**13**	**2**	**5**	**4**	**0**	**4**	**4**	**0**
FE	0.96	0.63	0.37	**20**	**1**	**4**	**3**	**1**	**5**	**7**	**0**
EP	0.80	0.82	0.84	0.92	**4**	**3**	**1**	**0**	**0**	**0**	**0**
OA	0.78	0.79	0.69	0.74	0.60	**11**	**5**	**0**	**2**	**3**	**0**
OB	0.88	0.75	0.70	0.77	0.80	0.41	**6**	**0**	**1**	**2**	**0**
BT	1.00	1.00	1.00	0.91	1.00	1.00	1.00	**2**	**0**	**0**	**0**
BR	1.00	0.79	0.74	0.67	1.00	0.81	0.88	1.00	**10**	**4**	**0**
Regab	0.96	0.84	0.80	0.64	1.00	0.80	0.84	1.00	0.72	**19**	**1**
Guiness	1.00	1.00	1.00	1.00	1.00	1.00	1.00	1.00	1.00	0.91	**4**

The Barbados Trench (BT) and Guiness (Gui) sites are not included in the hierarchical clustering, as shared only one species with other sites.

**Table 3:** Shared species among AEB regions.

Region	GoM	BAR	BR	GOG
**Depth**	350–3300 m	1300–2000 m	2200 m	3150 m
*Escarpia laminata*	X	X		
*B. boomerang complex*	X	X	X	X
*B. childressi complex*	X	X		X
*Calyptogena cf. kaikoi*	X	X		
*Phascolosoma turnerae*	X	X		
*Branchipolynoe seepensis*	X	X		X
*Bathynerita naticoidea*	X	X		
*Cataegis meroglypta*	X	X		
*Cordesia provannoides*	X			X
*Phymorhynchus cingulatus*	X		X	X
*Alvinocaris muricola*	X	X	X	X
*Munidopsis geyeri*	X			X
*Munidopsis livida*	X			X
*Munida microphtalma*	X	X		
*Chiridota heheva*	X		X	?
*Ophioctenella acies*	x	?	X	

The Bathymodiolinae species or complexes of species are the most widespread. The *B. boomerang* complex is found at the Florida escarpment site, the Blake Ridge diapir, the Barbados prism and the Regab site of Congo. The *B. childressi* complex is also widely distributed along the AEB from the Gulf of Mexico across to the Nigerian Margin, although not on the Regab or Blake ridge sites. The commensal polynoid, *Branchipolynoe seepensis* is another of the species shared by more than 2 regions (GoM, GoG and Barbados). Other species with distributions extending from the eastern to western Atlantic are: the gastropods *Phymorhynchus cingulatus* Warén & Bouchet, 2009, *Cordesia provannoides* Warén & Bouchet, 2009, the shrimp *Alvinocaris muricola* Williams, 1988, the galatheids *Munidopsis geyeri* Pequegnat & Pequegnat, 1970 and *Munidopsis livida* Perrier, 1886 and probably the holothurid *Chirodota heheva* Pawson & Vance, 2004.

**Figure 2:**
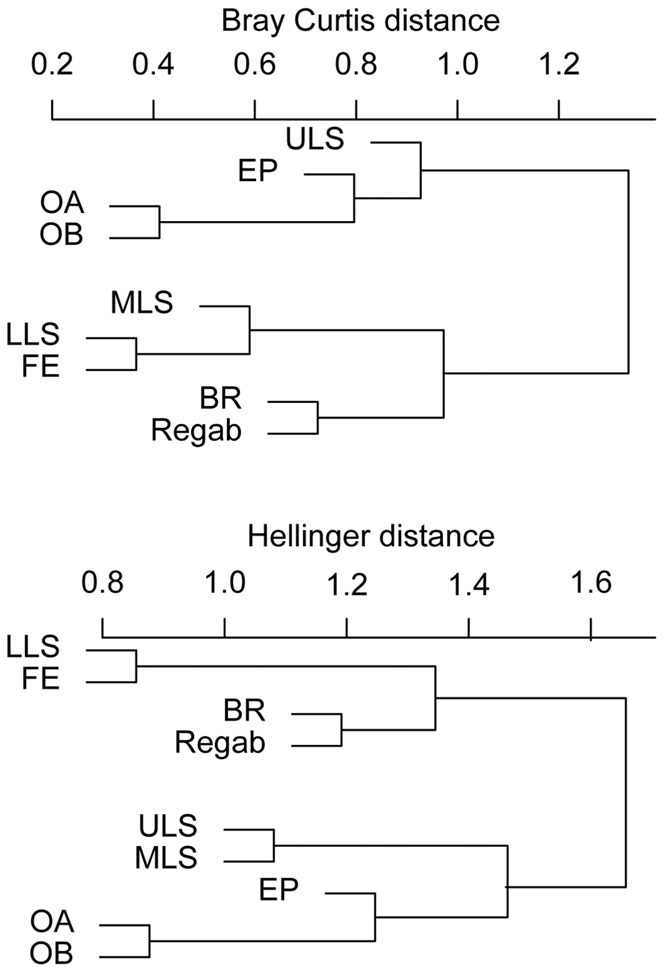
Hierarchical clustering of community similarity among AEB cold-seeps. Analysis based on species presence-absence at 9 AEB cold-seep sites including: the Louisiana Slope: ULS (<1000m), MLS (1000–2000m), LLS (>2000m), Florida Escarpment (FE, 3300m), south of Barbados prism: El Pilar (EP, 1300m), Orenoque A (OA, 1700m), Orenoque B (OB, 2000m), Blake ridge (BR, 2150m), Regab pockmark (3150m). The distance measuring dissimilarity between sites are the Bray Curtis distance (left) or Hellinger distance (right).

Similarity analyses performed using both distance indices (Bray Curtis and Hellinger), on the 9 sites indicated higher similarity among sites according to depth than to geographic distance (Table 2, Fig. 2). The Regab site clustered with the Blake Ridge diapir and with the deepest sites in the Gulf of Mexico (Florida Escarpment and lower Louisiana slope>2000m). The shallower Barbados prism communities (El Pilar 1300m, Orenoque 1700–2000m) grouped with shallow sites in the Gulf of Mexico: the upper and middle Louisiana slopes (Hellinger distance) or the upper slope only (Bray Curtis). The difference in the placement of the MLS is due to the inclusion of double absence by the Bray Curtis analysis and the exclusion of these data in the Hellinger analysis of similarity between samples. Indeed there are more ULS species absent from both MLS and LLS (11) than LLS species absent from MLS and ULS (7). Nevertheless the number of shared species between MLS and LLS is also higher (11) than between ULS and MLS (9) (Table 2), which is consistent with BC clustering.

**Figure 3:**
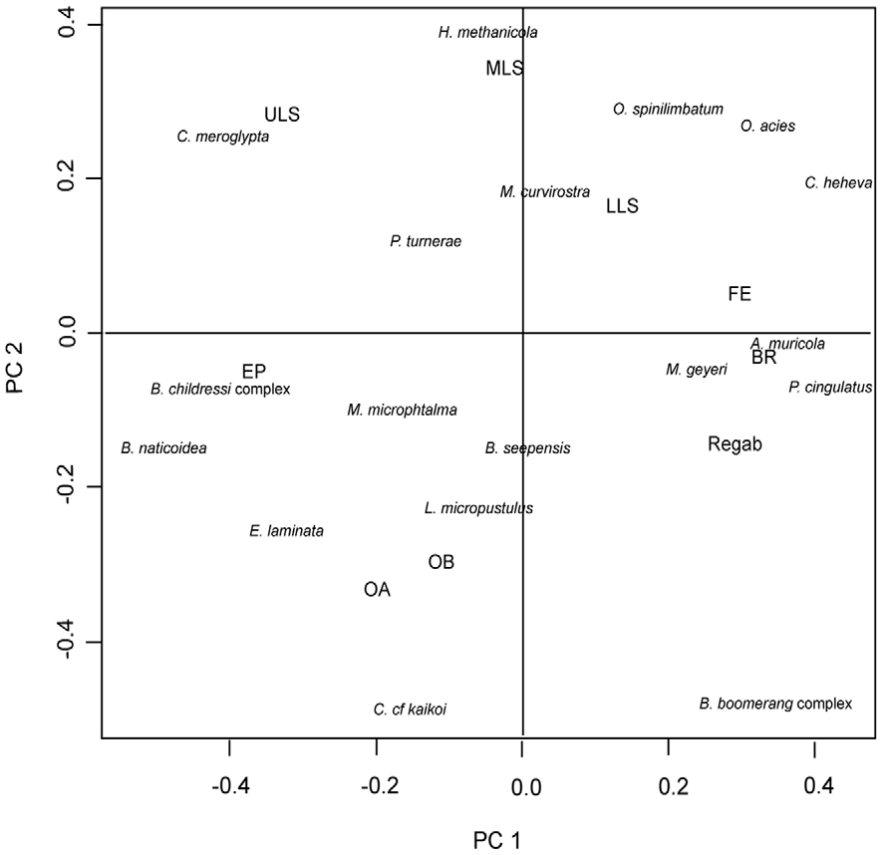
Principal Component Analysis of 9 AEB sites based on species presence/absence. Species data have been transformed using Hellinger distance. The first axis explains 24% of the variance, the second one 18%.

In the ordination of the 9 sites (PCA based on Hellinger distance) the sites were distributed along the first axis (24% of variance) primarily according to depth, with sites deeper than 2000m at the positive end of the axis and those <2000m at the negative one (Fig. 3). The second PCA axis (18%) separated the Gulf of Mexico sites from all other ones. The species that explain the majority of the variance along the first axis are, at the negative end (shallowest sites) the B. *childressi* complex, the gastropods *Bathynerita naticoidea* Clarke, 1989 and *Cataegis meroglypta* McLean & Quinn, 1987, *Phascolosoma turnerae* Rice, 1985 while *A. muricola*, *M. geyeri*, *C. provannoides*, *P. cingulatus*, *C. heheva* at the positive end separate the deepest sites towards the positive end of the axis. The GOM communities contain high abundance of *Hesiocaeca methanicola* Desbruyères & Toumond, 1998, *Munidopsis curviristra* Whiteaves, 1874 and *Ophienigma spinilimbatum* Stöhr & Segonzac, 2005 that are absent from other regions.

### Segregation by depth

Depth stratification in the west Atlantic cold-seep fauna is first observed at regional scale in the Gulf of Mexico with known sites and communities described all along the depth gradient analysed in the present study (500–3300m). It is also highlighted by the low number of shared taxa among the shallowest sites in the El Pilar region (1000–1300m) and those of the Orenoque A/B sectors (1700–2000m) and by the absence of any shared species with the deepest mud volcanoes located at 4900 m depth in the northern part of the prism (Table S1).

Depth boundaries seem to be particularly evident for the well sampled symbiont-bearing taxa. In the Gulf of Mexico and on the Barbados prism, the *B. childressi* complex of species occur at all shallower sites but are replaced at the deeper sites by *B. heckerae* observed up to 3300m. These two species have never been found to co-occur in the GoM and an additional species, *B. brooksi* is present at intermediate depths whose distribution overlaps with both *B. childressi* and *B. heckerae* at the two ends of its bathymetric range. However both species complexes have been found at intermediate depths between 1700 to 2200m in the Gulf of Mexico and on the Barbados prism. The same depth pattern is observed along the West Africa margins, where *B. boomerang* is the only species found at the Regab pockmark (3170m) while both species complexes co-occur at mid-depth sites along the Nigerian margin (1600 to 2200m). Empty shells of *B. childressi* and *B. boomerang* have also been sampled on a pockmark in the Congo basin at 1900m (unpubl. data). Vesicomyidae bivalve species are also segregated by depth within each region of the AEB. In the Gulf of Mexico, *Calyptogena ponderosa* Boss, 1968 and *Vesicomya cordata* Boss, 1968 are present on the upper and middle Louisiana slopes while another *Calyptogena* sp. occurs at the deeper Florida escarpment. Similarly, different species have been collected from the Barbados trench (*Calyptogena* sp.) compared to the nearby Orenoque/El Pilar shallower sites (*Laubiericoncha myriamae* Cosel & Olu, 2008), and from the Regab pockmark and the Guiness area (Table S1).

## Discussion

### Depth influence on cold-seep fauna composition

The analysis of cold-seep megafauna communities along the AEB revealed relatively high degrees of similarity among sites found in similar depth ranges, even across large geographic distances. The cold-seep sites in the eastern equatorial Atlantic shared almost 30% of the megafauna taxa with sites from similar depths in the western Atlantic, despite distance. Although few of the total number of examined species are shared among the sites, the similarity analysis based on their faunal composition, the Atlantic equatorial belt sites clustered within regions and according to depth ranges (Fig. 2), a result that is reinforced by the principal component analysis. These analyses suggest that seep community structure, at least for megafauna, is strongly controlled by depth, at the scale of the AEB.

In the analyses based on Hellinger distance, the depth separation between the two groups of sites is around 2000m. A second bathymetric segregation is observed on the PCA plot between 1300 and 1700m with the shallowest Barbados prism sites (El Pilar) clustering with the shallowest ones in the GoM (upper Louisiana slope). The highest species richness is observed at intermediate depths (between 1700 to 2000m) with species of both deeper and shallower communities. The differences among Bray-Curtis and Hellinger distance analyses suggest that there may be a gradual transition in this depth range and a mixing among the fauna of these two bathymetric zones between 1000 and 2000 m. In the study of west Atlantic cold-seep similarity [12], rapid replacement was also suggested at intermediate depths (1300–1700m). Similar patterns are found in the non-seep fauna of the Gulf of Mexico as well as in other ocean basins (e.g. [65], [66], [67]) with the maximum of alpha diversity at mid-slope depths (1500–2500m) for both mega- and macrofauna [68], [69]. Causes of depth zonation on continental margins have been attributed to physico-chemical parameters (temperature, water-masses, pressure), food availability, and biotic interactions (predation and competition) [70] and export POC flux was recently evidenced as the major factor explaining (macro-) faunal depth zonation patterns, as independent of the depth effect itself, for mid- or lower slopes in the GOM [71].

In contrast, export POC is not likely to be the main factor explaining depth patterns at seeps due to their primary reliance on local chemosynthetic productivity. Nevertheless, seep communities include non-endemic taxa, background species that are colonists (such as galatheids) or vagrant species (not included in this study) that would be expected to follow general depth zonation patterns driven by food availability, temperature, pressure or water masses. Additional depth-related factors structuring cold-seep communities include biotic interactions. Predation pressure is likely higher at the shallowest seeps [24], [27], [35] and may help explain differences observed between the shallow (above 1000m) and deep sites in Gulf of Mexico [40]. Similarly, large crustaceans (Lithodidae and Majidae) have been observed on the El Pilar mussel beds, as well as on the Guinesss pockmarks but not in the deepest sites of the Barbados prism or of the deep Gulf of Guinea.

The bathymetric zonation of the shallower *B. childressi* and the deeper *B. boomerang* species complexes of the AEB are more broadly supported by the global distributions of related bathymodiolin mussels. A combined phylogenetic analysis of three genes support the existence of a “*childressi*” clade including shallow water species from the west Pacific, while *B. heckerae* and B. *boomerang* cluster with the majority of the vent species that tend to live at deeper sites [12], [72]. Seep vestimentiferans tend to be more widely distributed than hydrothermal vent species, based on combined morphological descriptions and COI sequence data [19], and can have very large geographic ranges within bathymetric zones. Indeed the three described *Escarpia* species, are all very closely related morphologically and genetically [20], [73] but appear to be restricted to depths greater than 1300 m [19], [20]. Nevertheless, other siboglinids in the *Escarpia* clade, including *Seepiophila jonesi* and an undescribed *Escarpia* species, are found at shallower sites in the GoM and Atlantic seeps [36], [73]. Different vesicomyid species occur at shallow Louisiana slope sites and Florida escarpment [12]. Although depth ranges may be influenced by sampling bias, the six vesicomyid species described from seeps in the Gulf of Guinea seem to be distributed either shallower or deeper than 2000–2500m with a transition depth zone at in this depth range [60]. Apparent depth segregation of vesicomyids has been reported at seeps along other continental margins off Japan and Peru [5], [26]. *Calyptogena pacifica* occurs over a remarkably restricted depth range despite high dispersal capacities [23], with also a bathymetric segregation at the intra-specific level [74] like *B.childressi* [75].

It is important to remember that other seep environmental factors (geologic settings, seep chemistry and flow rates, substrate types) may vary with depth in the regions studied and, as discussed in the next section these factors are critical not only for the types of communities present, but even for the existence of seep macro- and megafauna. For example, fluid chemistry, depth related, was found to be a strong factor structuring distinct faunal islands (instead of biogeographical provinces), differing in species composition, along the Mid-Atlantic Ridge [3], [4].

### Variation within regions and depth ranges

**Figure 4:**
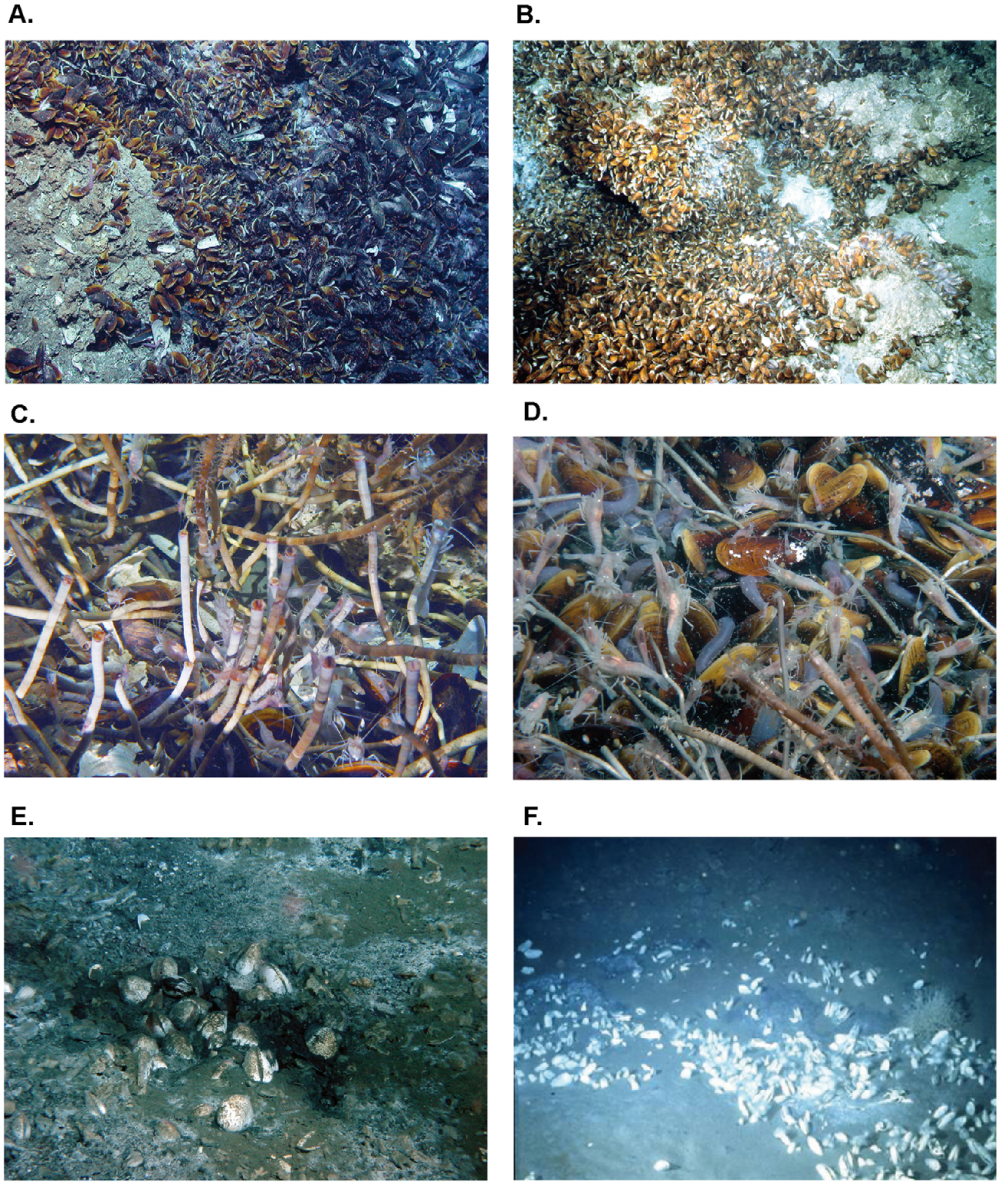
Similarities and differences among AEB cold-seep site habitats. A. Mussel bed (*Bathymodiolus brooksi*) from Atwater Valley at 2200m depth in the Gulf of Mexico, B. Mussel bed (*Bathymodiolus childressi*) on the El Pilar area, Barbados prism (1300m) (©Ifremer, Diapisub 1992), C. Mussels and tubeworms along with *Alvinocaris muricola* from Atwater Valley at 2200m depth in the Gulf of Mexico. (A. & C.: © MMS-NOAA OER Chemosynthetic Ecosystems study). D. *Bathymodiolus* aff. *boomerang* bed on the Regab pockmark off Congo associated with other amphi-Atlantic species (*Alvinocaris muricola*, *Chiridota* aff. *heheva*), and the Siboglinidae *Escarpia southwardae*. E. Vesicomyidae *Calyptogena valdiviae* and microbial mats on the Guiness area off Gabon (D. & E. ©Ifremer, Biozaire 2001). F. Vesicomyidae *Calyptogena aff. kaikoi* aggregate and sponges *Cladorhiza methanophila* in the Barbados trench. (©Ifremer, Manon 1992).

The present analysis provides evidence for depth as a structuring factor of AEB megafauna seep communities, but this factor only explains part (42%) of the variation in the communities observed in different areas. Local communities in the deep sea in general may be composed of species that exist as metapopulations whose regional distribution depends on a balance among global-scale, landscape-scale, and small-scale dynamics [76]. Variation in the type and magnitude of fluid venting will have a first order effect on presence of different types of seep foundation fauna and the level of chemoautotrophic primary production from both symbiont containing species and free living microbes in the site. Biogenic habitats created by microbial mats and the symbiotic species contribute to create heterogeneity in structural complexity (Fig. 4), habitat geochemistry, nutrient sources, and inter-specific interactions enhancing beta diversity of associated fauna [77].

In the Gulf of Mexico, over 90 seep sites have been visited by submersible [78]. Although broad similarities among sites within depth ranges were apparent in our analyses, individual sites within a depth range may vary with respect to the associated communities, the higher taxa of foundation fauna at species level (e.g. relative abundance of the different mussel species in a bed [40]). Some of these differences may be attributed to succession processes related to the age of the site and substratum evolution [79], the age of tubeworm aggregations [34], [37], and even this pathway of succession may differ from site to site [36].

As demonstrated for the export POC flux for detritus-based benthic communities [70], the fluid flow is also a limiting factor for seep communities. Methane and oxygen concentrations have been identified as important factors influencing the communities in seep mussel beds [33], [48], as has been sulphide concentration in tubeworm aggregations [37]. Highly variable methane concentrations above the pockmarks in the Gulf of Guinea ([80] and Charlou, Caprais, pers.com.) could explain the absence of any mytilid in the majority of explored pockmarks. The presence of multiple symbionts likely favours *B. heckerae*, *B. boomerang* and *B. brooksi* at sites where sulphide is more available than methane. This may convey competitive advantage to these species at some sites as observed for *B. heckerae* [81] or for *B. boomerang* which may be able to utilize reduced compounds from pore waters, by burrowing in sediments at low activity sites [44], [82]. Vesicomyid distribution is also likely to be influenced by specific adaptations to sulphide or oxygen concentrations, which can be correlated to methane fluxes in some environments [60], [83], [84], [85] and adaptations to specific geochemical environments has been suggested to have driven the evolution of co-existing vesicomyid genera [23]. Finally, the rare occurrence of Cladorhizidae sponges, in the Barbados trench [86] and on the Nigeria margin may be favoured by high methane fluxes but another unknown factor limits their distribution to other AEB seep sites. Further investigations of their habitat preferences and the environmental conditions at all of the sites are required to explain the distribution of these sponges.

### Potential for long distance larval dispersal

Considerable mixing among populations of vent organisms along ridge segments [22], and the lack of genetic structure that has been observed for numerous vent taxa over oceanic ridge scales [16] argue for long dispersal capacities of hydrothermal vent taxa. The relatively high degree of community similarity among AEB cold-seep regions suggests recent exchanges among the Gulf of Mexico, the Caribbean, and the Gulf of Guinea. The Blake Ridge diapir communities also share taxa with other regions but to a lower degree. Different deep and shallow currents have been suggested to provide connections for propagules between the Gulf of Mexico, the Barbados prism and Blake Ridge [12]. The longitudinal flow of the North Atlantic Deep Water, enhanced by equatorial intermediate jets, could theoretically provide a connection eastward to the west Africa margins or westward from these sites, but the relatively low velocities of these deep currents may not be sufficient to transport even long lasting propagules across the Atlantic [87]. Propagule transport by surface currents, which could produce crossing times of a few months, would be a more realistic time frame for many seep animals, assuming the larvae could persist at shallow depths.

Cold-seep amphi-Atlantic species represent various taxonomic groups differing in reproductive strategies. Possessing a planktotrophic larval form does not appear to be a prerequisite for long-distance dispersal, as lecitotrophic larvae may disperse over longer distance than planktotrophs in oligotrophic waters [88], [89]. Decrease of developmental and metabolic rates with temperature may also extend dispersal potential for lecitotrophic larvae in the cold deep sea [90]. Variable buoyancy of propagules can change the vertical dispersal of a larva in the water column (e.g. [91]). Thus, larvae can be transported by different water currents at different depths and subsequent divergent trajectories at different times during their development. Larvae of the gastropod *Cordesia provannoides*, or a very similar species, has been collected 0–100m below the surface in the tropical East Atlantic overlying a total water depth of 4570 m [59]. Teleplanic larvae (long-distance dispersing) have been demonstrated for several shallow water gastropods (e.g. [92]). Lengthy developmental period, long-lasting (>60 day) larvae and ontogenic vertical migration have also been shown for *Bathynerita naticoides* [93], found at both GOM and Barbados seeps. The gametogenic periodicity correlated with surface production demonstrated for *B. childressi* can enhance survival of its planktotrophic larvae and therefore long-range dispersal [94]. *B. childressi* larvae may be teleplanic and, according to known settlement times and spawning seasons, spend more than a year in the plankton [95] The small size of *Alvinocaris muricola* embryos also suggested planktotrophic larvae and the capacity for extended larval development [56], [96]. Consistently, phylogenetic analyses of the hydrothermal vent shrimps indicate that the *Alvinocaris* species do not cluster according to biogeographic regions [97].

Amphi-atlantic distribution at seeps also concerns taxa with potentially lecitotrophic larvae like the galatheid *M. geyeri* found at the deepest sites. Some galatheid species have very large egg sizes that apparently give them broad dispersal capabilities [98]. Several deep-sea galatheids appear to have an amphi-Atlantic distribution [57], as has been shown for a number of deep-sea decapods. However, additional genetic and larval biology studies are needed to verify these findings for the present species and understand the mechanisms sustaining this broad distribution. The giant isopod *Bathynomus giganteus* despite brooding eggs also has a broad distribution from GoM to GoG (Rowe pers com).

Genetic similarities between populations separated by long geographic distances may also result of low rates of evolution in the mitochondrial genes analysed rather than very recent genetic exchange between the various regions, hypothesis suggested for seep siboglinids [19], [99]. Although morphologically distinct, *E. southwardae*, *E. laminata* and *E. spicata* are genetically undistinguishable and could represent a single polymorphic species with extremely widespread distribution [20]. This is supported by a significant capacity for larval dispersal for *Escarpia* and *Lamellibrachia* with positively buoyant lecitotrophic larvae that can spend at least three weeks up in the water column [100]. However, there is unlikely to be significant gene flow between at least *E. laminata* and *E. spicata*, since the Panama Isthmus closure 3 million years ago. On the contrary, molecular analyses of Vesicomyidae suggest these clams have generally more restricted geographic and bathymetric distributions, and it has been suggested that an older radiation favoured higher diversification [101]. Geographic isolation but also physiological adaptations to geochemical environments are major factors for speciation within the genus *Calyptogena* [102]. Nonetheless, molecular studies support the view that some vesicomyid clams spread easily along continental margins and trans-Pacific migrations have been suggested for several species by molecular studies [103].

### Stepping stones?

More than 80% of Atlantic marine invertebrates that possess a planktotrophic larval form appear to have an amphi-Atlantic distribution, and 30% of molluscs of the eastern or western Atlantic are amphi-atlantic [104]. While some of these species may be capable of dispersing all the way across the Atlantic Ocean with long-distance planktonic larvae, other coastal species more likely use islands as ‘stepping stones’. For species associated with chemoautotrophic ecosystems, the occurrence of contemporary gene flow across the Atlantic equatorial belt via planktonic larvae could be sustained by larval exchanges along a continuum of seep sites, sunken wood and whale carcasses [52]. With the possible exceptions of the seep mussel commensal *Branchipolynoe seepensis*, which is also associated with the hydrothermal Bathymodiolinae all along the Mid Atlantic Ridge, and the shrimp *Alvinocaris muricola*, that may occur at the Logatchev vent site [55], the hydrothermal vent communities of the Mid Atlantic Ridge do not seem to serve as major stepping stones for AEB cold seep communities. A third species, the brittle star *Ophioctenella acies*, is shared between West Atlantic cold seeps and MAR vents, but has not yet been found at West Africa seeps.

Though some DNA evidence suggests that *B. heckerae* may have derived recently from *Bathymodiolus azoricus* [105], a recent phylogeny based on COI suggested colonization pathways of the seeps of the AEB appear distinct from those that led to the colonization of the MAR and the emergence of *B. azoricus* and *B. puteoserpentis* [52], and *B. brooksi* from the Gulf of Mexico appears to be basal to all of these groups [12]. Reports of shared vesicomyids among seeps on the West Florida escarpment, the Barbados accretionary prism and the Logatchev vent field on the Mid-Atlantic Ridge [11], [17] await further genetic investigations and the current revision of the vesicomyids [23], [54], [60]. Regardless, the MAR does not appear to be a consistent stepping stone for the fauna of the AEB seeps, but further exploration of low temperature vents or seeps and along transform faults may reveal potential favourable sites for seep taxa. In addition, as most of the known amphi-Atlantic AEB seep species are associated with the sites deeper than 2000m, investigations of the deeper MAR sites and transform faults, in particular the on the inter-tropical area, will provide important data to test the seep to vent stepping-stone hypothesis.

Sunken woods, whale carcasses or other sources of organic matter may also serve as stepping stones for seep species such as *C. provannoides* also found on wood falls [59]. Siboglinid tubeworms were brought to the surface from a shipwreck full of coffee beans and fruits lying at 1200 m of water off the north-western coast of Spain [106]. Clearly this is not the natural habitat for this seep species, however it demonstrates the ability of seep fauna to colonize diverse habitats where reducing chemicals are present in sufficient concentrations to support chemoautotrophic primary production.

### Conclusion, future directions

The similarity analyses presented in this paper suggest that the seep megafauna along the Atlantic equatorial belt do not primarily cluster according to biogeographic regions, as strongly structured by depth. This pattern is particularly evident for endemic seep fauna, and is supported by phylogenetic studies for some species. Different hypotheses may explain these broad geographic distributions, including present-day larval exchanges facilitated by extended larval durations of some seep taxa. Larval tracking in the vicinity of cold seep sites or along transects across the AEB could be used to address this hypothesis. It is also possible that many of the apparently shared species may in fact be cryptic species or species that are distinguishable morphologically but lack apparent genetic differentiation. The development of more molecular markers and population genetic studies are needed to better understand the genetic connections among regions and populations. A recent study demonstrated the power of nucleotide polymorphism in mitochondrial COI and coalescence analyses for tracing historical demographic events, genetic exchanges and population isolation in the case of hydrothermal vent species [107].

Exploration of new areas, such as the Amazon fan and other potential hydrocarbon seep areas off southern Brazil, potential seep sites off of the east coast of the U.S. and the Laurentian fan where chemosynthetic communities are known deeper than 3500m, and shallower sites in the Gulf of Guinea are need to further assess the role of depth as dominant factor structuring seep communities. Use of comparable sampling strategies and devices, increase of faunal collections in all regions, and collections of comparable environmental data sets are also needed to facilitate these comparisons and better understand the role of abiotic and biotic factors in structuring Atlantic cold-seep communities.

## Supporting Information

Table S1 List of macro- and megafaunal taxa identified in the AEB cold-seep sites. For abbreviations, see Table 1. Shared taxa are identified as followed: *: amphi-Atlantic species or species complex, ** species shared between at least 2 regions of the West A.
(0.51 MB DOC)
