# Supplementary material for: Biogeography and Potential Exchanges Among the Atlantic Equatorial Belt Cold-Seep Faunas
Source: PLoS One. 2010 Aug 5;5(8):e11967. doi: 10.1371/journal.pone.0011967 (PMC2916822; doi:10.1371/journal.pone.0011967)
Supplement: Table S1 — List of macro- and megafaunal taxa identified in the AEB cold-seep sites. For abbreviations, see Table 1. Shared taxa are identified as followed: *: amphi-Atlantic species or species complex, ** species shared between at least 2 regions of the West A. (0.51 MB DOC) [file pone.0011967.s001.doc]

List of macro- and megafaunal taxa identified in the AEB cold-seep sites. For abbreviations, see Table 1. Shared taxa are identified as followed: *: amphi-Atlantic species or species complex, ** species shared between at least 2 regions of the West Atlantic.

| **Site** |  | **ULS** | **MLS** | **LLS** | **FE** | **EP** | **OA** | **OB** | **BT** | **BR** | **REG** | **AST** | **HH, BH, WH** | **GUI** | **NIG** |
| --- | --- | --- | --- | --- | --- | --- | --- | --- | --- | --- | --- | --- | --- | --- | --- |
| **Symbiotic fauna** | Depth (m) | < 1000 | 1000-2000 | 2000-3000 | 3300 | 1300 | 1700 | 2000 | 4900 | 2200 | 3150 | 2800 | 3100 | 650 | 1800 |
| Porifera |  |  |  |  |  |  |  |  |  |  |  |  |  |  |  |
| Cladorhizidae | *Cladorhiza methanophila* |  |  |  |  |  |  |  | X |  |  |  |  |  |  |
|  | *gen.sp* |  |  |  |  |  |  |  |  |  |  |  |  |  | X |
| Hymedesmiidae | *Hymedesmia sp* | X |  |  |  |  |  |  |  |  |  |  |  |  |  |
| Annelida |  |  |  |  |  |  |  |  |  |  |  |  |  |  |  |
| Polychaeta |  |  |  |  |  |  |  |  |  |  |  |  |  |  |  |
| Siboglinidae | *Escarpia laminata* |  | X | X | X | X | X | X |  |  |  |  |  |  |  |
|  | *Escarpia southwardae* |  |  |  |  |  |  |  |  |  | X | X | ? |  |  |
|  | *Escarpia sp.* | X |  |  |  | X |  | X |  |  |  |  |  |  |  |
|  | *Lamellibrachia luymesi* |  | X | X |  |  |  |  |  |  |  |  |  |  |  |
|  | *Lamellibrachia sp.* |  | X | X |  | X |  |  |  |  |  |  |  |  |  |
|  | *Seepiophila jonesi* | X | X |  |  |  |  |  |  |  |  |  |  |  |  |
|  | *Bathymodiolus boomerang* |  |  |  |  |  | X | X |  |  |  |  |  |  |  |
| Mollusca | *B.* aff*. boomerang* |  |  |  |  |  |  |  |  |  | X |  |  |  |  |
| Bivalvia | *Bathymodiolus heckerae* |  |  | X | X |  |  |  |  | X |  |  |  |  |  |
| Bathymodiolinae | *B. boomerang* complex*** |  |  | X | X |  | X | X |  | X | X |  | ? |  | x |
|  | *Bathymodiolus brooksi* |  | X | X | X |  |  |  |  |  |  |  |  |  |  |
|  | *B. childressi* complex*** | X | X | X |  | X | X |  |  |  |  |  |  |  | x |
|  | *B. childressi* | X | X | X |  |  |  |  |  |  |  |  |  |  |  |
|  | *B. mauritanicus* |  |  |  |  |  |  |  |  |  |  |  |  |  | X |
|  | *B.* aff. *mauritanicus* |  |  |  |  | X | X |  |  |  |  |  |  |  |  |
|  | *Idas macdonaldi* | X |  |  |  |  |  |  |  |  |  |  |  |  |  |
|  | *Tamu fisheri* | X |  |  |  |  |  |  |  |  |  |  |  |  |  |
|  | *Calyptogena ponderosa* | X | X |  |  |  |  |  |  |  |  |  |  |  |  |
| Vesicomyidae | *Calyptogena cf. kaikoi*** |  |  |  | X |  |  |  | ? |  |  |  |  |  |  |
|  | *Calyptogena sp.* |  |  |  |  |  |  |  | X |  |  |  |  |  |  |
|  | *Laubiericoncha myriamae* |  |  |  |  |  | X | X |  |  |  |  |  |  |  |
|  | *Laubiericoncha chuni* |  |  |  |  |  |  |  |  |  | X | X | ? |  |  |
|  | *"Calyptogena" regab* |  |  |  |  |  |  |  |  |  | X | X | ? |  |  |
|  | *Elenaconcha guiness* |  |  |  |  |  |  |  |  |  |  |  |  | X |  |
|  | *Calyptogena valdiviae* |  |  |  |  |  |  |  |  |  |  |  |  | X |  |
|  | *Vesicomya chordata* | X |  |  |  |  |  |  |  |  |  |  |  |  |  |
|  | *Vesicomya cf. venusta* |  |  |  |  |  |  |  |  | X |  |  |  |  |  |
|  | *Lucinoma atlantis* | X |  |  |  |  |  |  |  |  |  |  |  |  |  |
| Lucinidae | *Lucinid spp.* | X |  | ? |  |  |  |  |  |  | X |  |  |  | x |
|  | *Acharax sp.* |  |  |  |  |  |  | X |  | X |  | X |  |  |  |
| Solemyidae | *gen sp* | X |  |  |  |  |  |  |  |  |  |  |  |  |  |
|  | *Thyasira oleophila* | X |  |  |  |  |  |  |  |  |  |  |  |  |  |
| Thyasiridae | *Thyasira sp.* |  |  |  |  | X |  |  |  |  | X |  |  |  |  |
|  |  |  |  |  |  |  |  |  |  |  |  |  |  |  |  |
| **Associated Fauna** |  |  |  |  |  |  |  |  |  |  |  |  |  |  |  |
| Protozoa |  |  |  |  |  |  |  |  |  |  |  |  |  |  |  |
| Xenophyophora | *Syringammina sp.* |  |  |  |  |  |  |  |  | X |  |  |  |  |  |
| Porifera | *Geodia sp.* |  |  |  |  |  | X |  |  |  |  |  |  |  | X |
| Actinaria | *Monactis vestita* |  |  |  |  |  |  |  | X |  |  |  |  |  |  |
|  | *gen. sp.* |  |  | X | X |  | X | X |  | X | X |  |  |  |  |
| Sipuncula | *Phascolosoma turnerae*** | X | X | X |  |  |  |  |  |  |  |  |  |  |  |
|  | *Phascolosoma cf turnerae* |  |  |  |  |  |  | X |  |  |  |  |  |  |  |
|  | *gen. sp.* |  |  |  |  |  |  |  |  | X |  |  |  |  |  |
| Annelida |  |  |  |  |  |  |  |  |  |  |  |  |  |  |  |
| Polychaeta |  |  |  |  |  |  |  |  |  |  |  |  |  |  |  |
| Amphinomidae | *Eurythoe n. sp. nov.* | X | X | X |  |  |  |  |  |  |  |  |  |  |  |
| Ampharetidae | *gen.sp.* |  |  |  | X |  |  |  | ? |  | X |  |  |  |  |
|  | *Amythasides sp.* |  |  |  | X |  |  |  |  |  |  |  |  |  |  |
|  | *Glyphanostonum sp.* |  |  |  | X |  |  |  |  |  |  |  |  |  |  |
| Capitellidae | *gen. sp.* | ? | X | X | X |  |  |  |  | X | X |  |  |  |  |
| Chaetopteridae | *gen. sp.* |  |  | X |  |  |  |  | ? | X |  |  |  |  |  |
| Cossuridae |  |  |  |  |  |  |  |  |  |  | X |  |  |  |  |
| Dorvilleidae |  |  |  |  | X |  |  |  |  |  | X |  |  |  |  |
| Polynoidae | *Branchinotogluma sp. nov.* | ? | ? | X | X |  |  |  |  |  |  |  |  |  |  |
|  | *Branchipolynoe seepensis** | X | X | X | X |  | X | X |  |  | X |  |  |  | ? |
|  | *Harmothoe n sp* | X | X | X |  |  |  |  |  |  |  |  |  |  |  |
|  | *gen. sp.* | ? |  | ? | X |  |  |  |  |  |  |  |  |  |  |
| Flabelligeridae | *Flabelligera sp.* |  | X | X | X |  |  |  |  |  |  |  |  |  |  |
| Hesionidae | *Hesiocaeca methanicola* | X | X | X |  |  |  |  |  |  |  |  |  |  |  |
| Hesionidae | *gen.sp.* |  |  | X | X |  |  |  |  |  | X |  |  |  |  |
| Maldanidae | *gen.sp.* |  | X | X |  |  |  |  | ? | X |  |  |  |  |  |
|  | *Nicomache sp.* | ? | X | X | X |  |  | X | ? | ? |  |  |  |  |  |
| Nautininiellidae | *Vesicomyicola trifurcatus* |  |  |  |  |  |  |  |  | X |  |  |  |  |  |
|  | *gen. sp.* |  |  | X | X | X | X | X | X |  |  |  |  |  |  |
| Nereidae | *Nereis n. sp.* | X |  |  | X |  |  |  |  |  |  |  |  |  |  |
| Orbiniidae | *Scoloplos sp.* |  |  |  | X |  |  |  |  |  |  |  |  |  |  |
|  | *Methanoaricia dendrobranchiata* | X |  |  |  |  |  |  |  |  |  |  |  |  |  |
| Pilargidae | *Synelmis sp.* |  |  |  | X |  |  |  |  |  |  |  |  |  |  |
|  | *gen. sp.* |  |  |  |  |  |  |  |  |  | X |  |  |  |  |
| Phyllodocidae | *Protomystides sp.* |  | X | X | X |  |  |  |  |  |  |  |  |  |  |
| Sabellidae | *gen. sp.* |  |  |  | X |  |  |  |  |  |  |  |  |  |  |
| Serpulidae | *gen. sp.* | ? |  |  |  |  | X |  |  |  |  |  |  |  |  |
| Spionidae | *gen. sp.* |  |  |  | X |  |  |  |  |  | X |  |  |  |  |
|  | *Prionospio sp.* |  |  | X |  |  |  |  |  |  |  |  |  |  |  |
| Syllidae | *gen. sp.* |  |  |  |  | X |  |  |  |  |  | X |  |  |  |
| Mollusca |  |  |  |  |  |  |  |  |  |  |  |  |  |  |  |
| Polyplacophora | *Leptochiton spp.* | ? |  |  |  |  |  |  |  |  |  |  |  |  |  |
|  | *Leptochiton micropustulus* |  |  |  | X |  |  |  |  |  |  |  |  |  |  |
| Aplacophora | *gen. sp.* |  |  |  | X |  |  |  |  |  |  |  |  |  |  |
| Gastropoda |  |  |  |  |  |  |  |  |  |  |  |  |  |  |  |
| Buccinidae | *Buccinum galetae* | X |  |  |  |  |  |  |  |  |  |  |  |  |  |
| Cancellariidae | *Cancellaria rosewateri* | X |  |  |  |  |  |  |  |  |  |  |  |  |  |
| Capulidae | *Hyalorisia galea* | X |  |  |  |  |  |  |  |  |  |  |  |  |  |
| Conidae | *Phymorhynchus cingulatus* |  |  |  |  |  |  |  |  |  | X |  |  |  |  |
|  | *Phymorhynchus* aff*. cingulatus** |  |  |  | X |  |  |  |  | X |  |  |  |  |  |
|  | *Phymorhynchus* aff *. alberti* |  |  |  | X |  |  |  |  |  |  |  |  |  |  |
|  | *Phymorhynchus n. sp.* |  |  | X | X |  |  |  |  |  |  |  |  |  |  |
|  | *Phymorhynchus coseli* |  |  |  |  |  |  |  |  |  | X |  |  |  |  |
|  | *Phymorhynchus sp.* |  |  |  |  |  |  | X | X |  |  |  |  |  |  |
|  | *gen. sp.* | ? |  |  |  |  | X |  |  |  |  |  |  |  |  |
| Hyalogirinidae | *Hyalogyrina rissoella* |  |  |  |  |  |  |  |  |  | X |  |  | X |  |
| Lepetodrilidae | *Lepetodrilus shannonae* |  |  |  |  |  |  |  |  |  | X |  |  |  |  |
|  | *Lepetodrillus n. sp* |  |  | X | X |  |  |  |  |  |  |  |  |  |  |
|  | *Leptogyra costellata* |  |  |  |  |  |  |  |  |  | X |  |  |  |  |
|  | *Leptogyra n. sp.* |  |  |  |  |  |  |  |  | X |  |  |  |  |  |
| Neritidae | *Bathynerita naticoidea*** | X |  |  |  | X | X |  |  |  |  |  |  |  |  |
| Neolepetopsidae | *Paralepetopsis floridensis* |  |  |  | X |  |  |  |  |  |  |  |  |  |  |
| Provannidae | *Provanna reticulata* |  |  |  |  |  |  |  |  |  | X |  |  |  |  |
|  | *Provanna chevalieri* |  |  |  |  |  |  |  |  |  | X |  |  |  |  |
|  | *Provanna sculpta* | X | X |  |  |  |  |  |  |  |  |  |  |  |  |
|  | *Cordesia provannoides** |  |  |  | X |  |  |  |  |  | X |  |  |  |  |
| Pyropeltidae | *Pyropelta oluae* |  |  |  |  |  |  |  |  |  | X |  |  |  |  |
|  | *Paralepetopsis sasakii* |  |  |  |  |  |  |  |  |  | X |  |  |  |  |
|  | *Paraleptopsis sp.* |  | X | X |  |  |  |  |  |  |  |  |  |  |  |
| Trochidae | *Gaza fisheri* | X |  |  |  |  |  |  |  |  |  |  |  |  |  |
|  | *Cataegis meroglypta*** | X | X |  |  | X |  |  |  |  |  |  |  |  |  |
|  | *Pyropelta sibuetae* |  |  |  |  |  |  |  |  |  |  |  |  | X |  |
|  | *Pyropelta sp.* |  | X | X | X |  |  |  |  | ? |  |  |  |  |  |
| Trophoninae | *gen.sp.* |  |  |  |  | X |  | X |  |  |  |  |  |  |  |
| Turbinidae | *Cantrainea macleani* | X |  |  |  |  |  |  |  |  |  |  |  |  |  |
|  | *Fucaria n. sp.* |  |  | X | X |  |  |  |  |  |  |  |  |  |  |
| Bivalvia |  |  |  |  |  |  |  |  |  |  |  |  |  |  |  |
| Cuspidaria |  |  |  | X | X |  |  |  |  |  |  |  |  |  |  |
| Arthropoda |  |  |  |  |  |  |  |  |  |  |  |  |  |  |  |
| Crustacea |  |  |  |  |  |  |  |  |  |  |  |  |  |  |  |
| Alvinocarididae | *Alvinocaris muricola** |  | X | X | X | ? | X | ? |  | X | X |  | X |  | ? |
|  | *Alvinocaris stactophila* | X |  |  |  |  |  |  |  |  |  |  |  |  |  |
|  | *Alvinocaris methanolphila* |  |  |  |  |  |  |  |  | X |  |  |  |  |  |
|  | *Alvinocaris sp.* |  |  |  |  |  |  |  |  | X |  | X |  |  |  |
| Galatheidae | *Munidopsis geyeri** |  |  |  | X |  |  |  |  |  | X |  |  |  |  |
|  | *Mundopsis hirtella* |  |  |  |  |  |  |  |  |  | X |  |  |  |  |
|  | *Munidopsis livida** |  |  | X |  |  |  |  |  |  | X |  | X |  |  |
|  | *Munidopsis sp.* | X |  | X | X |  | X | X | X | X |  | X |  |  |  |
|  | *Munida microphtalma*** | X |  |  |  |  | X |  |  |  |  |  |  |  |  |
|  | *Munidopsis sp 1* | X | X |  |  |  |  |  |  |  |  |  |  |  |  |
|  | *Munidopsis sp 2* | X |  |  |  |  |  |  |  |  |  |  |  |  |  |
|  | *Munidopsis* aff. *bracteosa* |  |  | X |  |  |  |  |  |  |  |  |  |  |  |
|  | *Munidopsis* aff*. cascadia* |  |  | X |  |  |  |  |  |  |  |  |  |  |  |
|  | *Munidopsis curvirostra* |  | X |  |  |  |  |  |  |  |  |  |  |  |  |
|  | *Munidopsis* aff. *segonzaci* |  |  | X |  |  |  |  |  |  |  |  |  |  |  |
|  | *Munidopsis* aff*. simils* |  | X | X |  |  |  |  |  |  |  |  |  |  |  |
| Lithodidae | *Paralomis arethusa* |  |  |  |  |  | X |  |  |  |  |  |  |  |  |
|  | *Paralomis cubensis* | X |  |  |  |  |  |  |  |  |  |  |  |  |  |
|  | *Lithodes mannaingi* |  |  |  |  | X |  |  |  |  |  |  |  |  |  |
|  | *Lithodes mannaingi* |  |  |  |  | X |  |  |  |  |  |  |  |  |  |
| Echinodermata |  |  |  |  |  |  |  |  |  |  |  |  |  |  |  |
| Holothuroidea |  |  |  |  |  |  |  |  |  |  |  |  |  |  |  |
| Synaptidae | *Chiridota heheva** |  | X | X | X |  |  |  |  |  |  |  |  |  |  |
|  | *Chiridota* aff. *heheva* |  |  |  |  |  |  |  |  | x | X | X | X |  | ? |
|  | *Chiridota sp.* |  |  |  |  |  |  |  |  | X |  |  |  |  |  |
| Synallactidae | *gen. Sp.* | X |  |  |  |  |  |  |  |  |  |  |  |  |  |
| Ophiuroidea |  |  |  |  |  |  |  |  |  |  |  |  |  |  |  |
| Ophiacanthidae | *Ophienigma spinilimbatum* |  | X | X | X |  |  |  |  |  |  |  |  |  |  |
| Ophiuridae | *Ophioctenella acies* |  | X | X | X |  |  | ? |  | X |  |  |  |  |  |
| Asteroidea |  |  |  |  |  |  |  |  |  |  |  |  |  |  |  |
| Goniasteridae | *Ceramaster granularis* |  |  |  |  |  | X |  |  |  |  |  |  |  |  |
|  | *Plinthaster perrieri* |  |  |  |  |  | X |  |  |  |  |  |  |  |  |
|  | *Scleracterias tanneri* | X |  |  |  |  |  |  |  |  |  |  |  |  |  |
| Echinoidea | *Sarsiaster griegi* |  |  |  |  |  |  |  |  | X | ? |  |  |  |  |
| Chordata |  |  |  |  |  |  |  |  |  |  |  |  |  |  |  |
| Zoarcidae | *Pachycara sulaki* |  |  |  | X |  |  |  |  |  |  |  |  |  |  |
|  | *Thermaces pelophilum* |  |  |  |  |  | X | X |  |  |  |  |  |  |  |
|  | *Lycodes sp.* |  |  |  |  |  |  |  |  |  | X |  |  |  |  |
